# Increased expression of MNK1b, the spliced isoform of MNK1, predicts poor prognosis and is associated with triple-negative breast cancer

**DOI:** 10.18632/oncotarget.24417

**Published:** 2018-02-05

**Authors:** Celia Pinto-Díez, Eva M. García-Recio, M. Isabel Pérez-Morgado, Marta García-Hernández, Lara Sanz-Criado, Silvia Sacristán, M. Val Toledo-Lobo, Belén Pérez-Mies, Isabel Esteban-Rodríguez, Alejandro Pascual, Mercedes Garcia-Villanueva, Noelia Martínez-Jañez, Víctor M. González, M. Elena Martín

**Affiliations:** ^1^ Servicio de Bioquímica-Investigación, IRYCIS-Hospital Ramón y Cajal, Madrid, Spain; ^2^ Departamento de Biomedicina y Biotecnología, Universidad de Alcalá, Alcalá de Henares, Madrid, Spain; ^3^ Servicio de Anatomía Patológica, IRYCIS-Hospital Ramón y Cajal, Madrid, Spain; ^4^ Sevicio de Anatomía Patológica, IDYPAZ-Hospital La Paz, Madrid, Spain; ^5^ Servicio de Oncología, IRYCIS-Hospital Ramón y Cajal, Madrid, Spain

**Keywords:** breast cancer, MNK1, molecular tumor type, pronostic marker, triple-negative

## Abstract

MAP kinase interacting kinases (MNKs) modulate the function of oncogene eukaryotic initiation factor 4E (eIF4E) through phosphorylation, which is necessary for oncogenic transformation. MNK1 gives rise to two mRNAs and thus two MNK1 isoforms, named MNK1a and MNK1b. MNK1b, the splice variant of human MNK1a, is constitutively active and independent of upstream MAP kinases. In this study, we have analyzed the expression of both MNK1 isoforms in 69 breast tumor samples and its association with clinicopathologic/prognostic characteristics of breast cancer. MNK1a and MNK1b expression was significantly increased in tumors relative to the corresponding adjacent normal tissue (*p* < 0.001). In addition, MNK1b overexpression was found in most of the triple-negative tumors and was associated with a shorter overall and disease-free survival time. Overexpression of MNK1b in MDA-MB-231 cells induced an increase in the expression of the MCL1 antiapoptotic protein and promoted proliferation, invasion and colony formation. In conclusion, a high expression level of MNK1b protein could be used as a marker of poor prognosis in breast cancer patients and it could be a therapeutic target in triple-negative tumors.

## INTRODUCTION

Breast cancer has an heterogeneous nature [1]. Breast tumors can be classified into subtypes based on their expression profile, and each subtype is associated with distinct histological markers and clinical parameters. The molecular classification is based on the presence of the hormone receptors (estrogen receptor [ER] and/or progesterone receptor [PR]), the human epidermal growth factor receptor 2 (HER2) and on the epithelial cell of origin (luminal and basal-like). Each subtype has a different prognosis and treatment response: thus, because ER is a therapeutic target, luminal subtypes are amenable to hormone therapy and, in consequence, are associated with less aggressive metastatic disease and longer disease-free survival [2]. Similarly, the HER2 group is a potential candidate for trastuzumab therapy. However, the absence of expression of a known therapeutic target makes triple-negative tumors more biologically aggressive, difficult to treat, and often have a poor prognosis.

The MAP kinase interacting kinase 1 (MNK1) has been shown to bind to and to be a substrate for both the mitogen-activated extracellular signal-regulated protein kinases (ERK) 1/2 as well as the stress-activated p38 MAP kinases (p38 MAPKs) both *in vitro* and *in vivo* [3]. MNK1b is a truncated isoform lacking the MAP kinase-binding motif present in the C-terminal region of MNK1a, which arises from an alternatively spliced transcript of the MNK1 gene [4]. Both MNK1 isoforms have a nuclear localization signal (NLS) in the N-terminal region that allows the kinase to enter into the nucleus [5]. MNK1a contains a nuclear export signal (NES) that ensures its cytoplasmic localization [5], whereas MNK1b lacks this NES, being cytoplasmic and nuclear [4]. From the different features in human MNK1 isoforms, we have shown that MNK1b has higher basal activity than MNK1a. Moreover, MNK1b activity does not correlate with the phosphorylation of the activation loop residues and seems to be independent of the upstream kinases (ERK1/2 and p38 MAP kinase) [4].

The only well-characterized substrate for MNK1 is the eukaryotic initiation factor 4E (eIF4E) [6]. It has been shown that eIF4E overexpression in a variety of cancers including breast, head and neck, colon, prostate, kidney and lung is related to disease progression [7–9]. Elevated levels of phosphorylated eIF4E are found in human cancer tissues obtained from patients with lung, head, colorectal, and gastric cancers and primary pancreatic ductal adenocarcinoma [10, 11]. Nuclear eIF4E phosphorylation appears to be important to control the transport of cyclin D1 mRNA and for the transforming properties of eIF4E [12]. Other studies have established that phosphorylation of eIF4E on Ser209 by MNK1/2 is an absolute requirement for the oncogenic action of eIF4E. The inhibition of MNK activity with different MNK1 inhibitors reduces several tumoral characteristics such as proliferation, colony formation or migration in human breast cell lines [13–15]. On the other hand, overexpression of the oncogene HMD2 in cancer cells is regulated by eIF4E, so that the overexpression of eIF4E promotes the export of the HDM2 mRNA in a MAP kinase- and Mnk1-dependent manner [16]. In addition, Wendel and colleagues [17] have shown that overexpression of a constitutively active MNK1 diminishes the apoptosis and accelerates the development of tumors in an experimental model of mice while an inactive mutant reduces the development of these tumors. Finally, it has been demonstrated that eIF4E is completely dephosphorylated in MNK1 and MNK2 knock-out mice, which do not exhibit any apparent phenotypic consequences, questioning a vital role of MNK activity [18].

This study aimed to determine the levels of MNK isoforms in breast cancer, whether they are associated with clinicopathological data and whether the constitutively active MNK1b is differentially expressed relative to MNK1a. Interestingly, we observed increased MNK1a and MNK1b levels in breast tumors and that MNK1b expression correlated with some aspects of clinicopathological status, especially with triple–negative tumors. In addition, the role of the overexpression of MNK1a or MNK1b on oncoprotein expression, migration, invasion and proliferation in triple negative breast cell lines has been investigated.

## RESULTS

### MNK1a/b levels are increased in breast cancer tissues

The expression of MNK1a and MNK1b was analyzed in 69 tumor samples from female patients diagnosed with breast cancer and the corresponding adjacent normal tissue. The patient’s characteristics are shown in Table 1. Most of the patients (70%) were older than 50 years, with a median age of 67 (range, 33–96 years). The pathologic tumor stage was predominantly T2–T4 (72.5%) and more of the 50% of the tumors showed positive nodal status. Almost all tumors showed histological grade 2 and 3 (45 and 48%) and only 15% of the cases showed grade 1.

**Table 1:** Patients’s characteristics included in the study

Variables	Number of patients%)
all	69 (100)
Age at surgery	
<50	20 (29)
>50	48 (69.6)
ND	1 (1.4)
pT	
pT1	16 (23.2)
pT2-T4	50 (72.5)
ND	3 (4.3)
Nodal status	
Negative	24 (34.8)
Positive	39 (56.5)
ND	6 (8.7)
Tumor grade	
G1	10 (14.5)
G2	31 (44.9)
G3	26 (37.7)
ND	2 (2.9)
Molecular types	
Luminal	39 (56.6)
TN	15 (21.7)
HER2	15 (21.7)
ER	
Negative	24 (34.7)
positive	45 (65.2)
PR	
Negative	28 (40.6)
positive	41 (59.4)
HER2	
Negative	54 (78.3)
positive	15 (21.7)
Ki-67 level	
<15%	32(46.4)
>15%	35 (50.7)
ND	2 (2.9)

ER, Estrogen receptor; PR, Progesterone receptor;

pT, Pathologic tumor stage; ND, not determined.

Breast cancer molecular subtypes were classified according following criteria:

Luminal: ER+ and/or PR+, HER2–

Triple-negative (TN): ER–, PR–, HER2–

HER2 positive: ER–/+, PR–/+, HER2+

Thirty nine patients were Luminal, 15 triple-negative (TN) and 15 HER2 positive. Hormone receptors, estrogen and progesterone, were expressed in 45 and 41 patients, respectively (65.2 and 59.4%).

**Figure 1:**
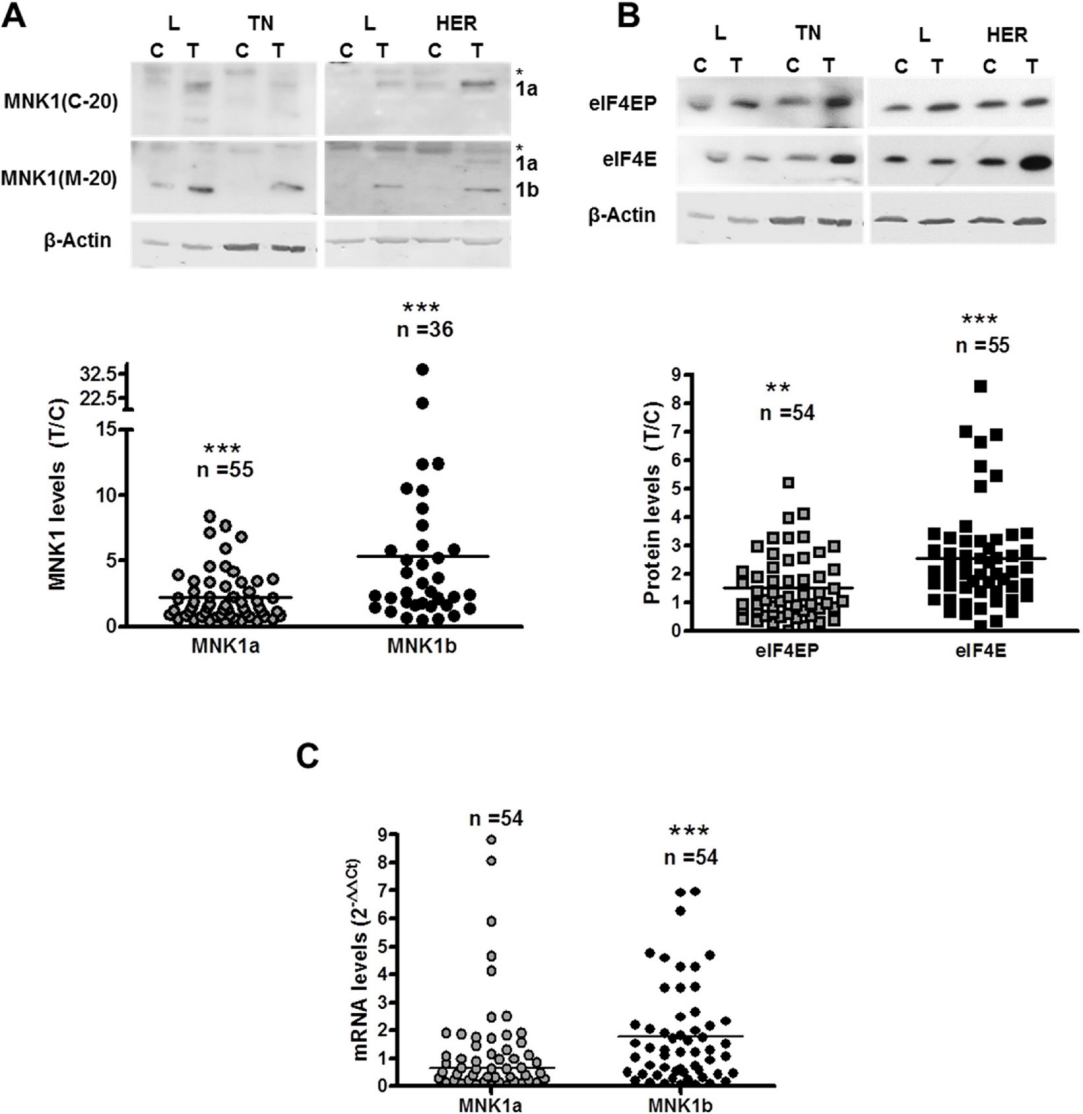
MNK1 isoforms, total and phosphorylated eIF4E levels in breast cancer samples Western blot analysis of MNK1a and MNK1b (**A**) and phosphorylated and total eIF4E (**B**) was done as described in Materials and Methods section. The amount of β-actin was used as a control for the homogeneity of loading. Scatter plots represent the quantification of protein levels normalized with respect to actin levels and expressed as the ratio (T/C) between tumor sample (T) and normal adjacent sample (control,C). A single point represents one sample analyzed. On the top of the Scatter plot “n” indicated the number of samples analyzed. Representative blots are shown on the top of the figure. L: Luminal; TN: Triple-negative; HER: HER2 positive. ^*^in (A) unspecific band. (**C**) Quantitative RT-PCR analysis of MNK1a and MNK1b (normalized to PGK expression) was done as described in Materials and Methods section. Scatter plots represent the quantification of each samples analyzed and horizontal bar indicates the average. Statistical differences relative to 1; ^**^p < 0.01; ^***^p < 0.001.

The expression of the two isoforms of MNK1, MNK1a and MNK1b, was analyzed on western blots, and the ratio between tumor (T) and adjacent normal (control, C) tissues was calculated. We used two specific antibodies, anti MNK1(C-20) generated against a C-terminal peptide which only recognized MNK1a isoform, and an antibody raised against a peptide mapping at the N-terminus of Mnk1 of mouse origin (MNK1 (M-20)) that recognizes both isoforms of human MNK1, although experimental results in our laboratory indicated that it recognizes MNK1b with higher affinity than MNK1a in western blot. As shown in Figure 1A, the levels of MNK1a and MNK1b increased significantly in tumor tissue relative to control tissue (about 2.2 and 5.3-fold, respectively). In addition, we analyzed the phosphorylation status and the expression of eIF4E, as a substrate of MNK1a and MNK1b (Figure 1B). As expected, phosphorylation and levels of eIF4E increased significantly in the tumor samples (about 1.5 and 2.6-fold respectively), what is in agreement with the results shown by other authors [7, 10, 19].

To check if the changes in the MNK1b levels were due to changes in the mRNA levels, a quantitative analysis of the mRNAs of MNK1a and MNK1b was performed as described in Materials and Methods section. The RNA quality was analyzed using an Experion Station. We used samples with similar RQI factors (RNA quality index) between control and tumor tissues (54 samples). The levels of MNK1a isoform did not change whereas MNK1b mRNA levels increased significantly (Figure 1C). Interestingly, MNK1b protein and mRNAs levels correlated significantly (Spearman *r* = 0.403, *p* = 0.003) and MNK1a/Mnk1b mRNAs correlated inversely (spearman *r* = –0.417, *p* = 0.002) indicating that the switch in the ratio of MNK1b/MNK1a mRNAs probably by alternative splicing is responsible of the increased MNK1b expression. Therefore, one can conclude that the increase in MNK1b levels result from an increase in its mRNA expression.

### Increased MNK1b levels are associated with clinicopathological status

Next, we analyzed whether the expression of MNK1 isoforms, and the expression and phosphorylation of eIF4E correlate with clinical-pathologic characteristics of the tumors (Supplementary Table 1). No significant correlation was found between MNK1 isoforms expression, eIF4E expression or its phosphorylation status with almost none of the parameters studied. We only found that MNK1a decreased significantly in those tumors positive for Progesterone receptor, MNK1b is higher in pT1 tumors than in pT2-4 tumors and eIF4E levels increased significantly in positive node tumors. Taken into account that the ratio T/C for MNK1b could not be calculated in many cases (>47%) because the protein was undetectable in control tissues by western blot analysis, we decided to perform an additional qualitative analysis. A score of 0 was assigned when no changes in MNK1b expression levels between tumor and control tissue were observed or when the protein was not detected in tumor tissue. A score of 1 was given when MNK1b levels were higher in tumors than in adjacent normal tissue or when MNK1b was only detected in tumor tissue. Higher MNK1b levels were found in 62% of the tumor samples (41/66). As shown in Table 2, there are significant differences in the overexpression of MNK1b between different molecular types, almost all TN tumors (93%) are positive for MNK1b expression. In addition, there are differences although not statistically significant (*p* = 0.094) in the overexpression of MNK1b between HER2-negative and HER2-positive tumors (67% vs. 43%). It should be noted that, as MNK1a (Supplementary Table 1), MNK1b is overexpressed in tumors negative for progesterone receptor (77% *vs.* 52% in positive PR, *p* = 0.046).

**Table 2:** MNK1b overexpression and clinical-pathologic characteristics

Variables	MNK1b overexpression			chi-square	*P* value
**Age at surgery**	***N***	**Cases positives**	**%**		
<50	21	13	62	0.101	0.751
>50	46	28	61		
**pT**					
pT1	15	11	73	1.090	0.296
pT2–T4	48	28	58		
**Nodal status**					
Negative	21	13	62	0.049	0.825
Positive	39	23	59		
**Tumor grade**					
G1	10	5	50	3.522	0.172
G2	31	23	74		
G3	23	12	52		
**Molecular types**					
Luminal	39	23	59	7.406	0.025^*^
TN	13	12	92		
HER2	14	6	43		
**ER**					
Negative	21	15	71	1.130	0.287
positive	45	26	58		
**PR**					
Negative	26	20	77	3.994	0.046^*^
positive	40	21	52		
**HER2**					
Negative	52	35	67	2.802	0.094
positive	14	6	43		
**Ki-67 level**					
<15%	31	22	71	1.839	0.175
>15%	33	18	55		

^*^p < 0.05 Chi-square test.

**Figure 2:**
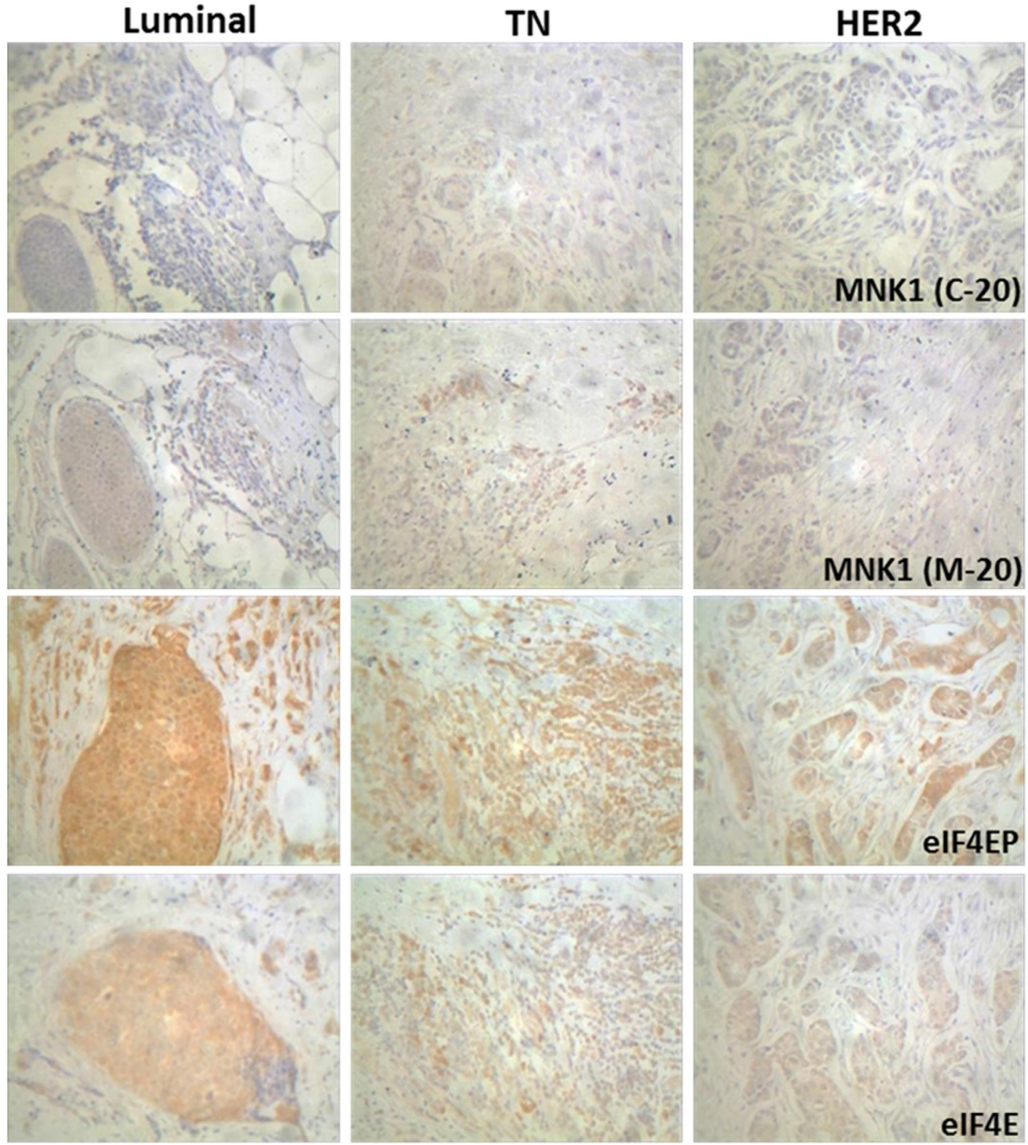
Immunohistochemical staining of MNK1 isoforms, total and phosphorylated eIF4E levels in breast cancer samples Immunohistochemistry was performed using anti-MNK1 (C-20), MNK1 (M-20), eIF4E(Ser209)P and eIF4E antibodies as described in Materials and Methods section. Representative tumor sections of each molecular types are shown (200×).

The levels of MNK isoforms, phosphorylated eIF4E and eIF4E were also detected by immunohistochemical staining in 34 paraffin sections (18 Luminal, 7 TN and 9 HER2 positive) as described in Materials and Methods section. The results of the immunohistochemical stainings are shown in Figure 2 and Table 3. MNK1a and MNK1b positive staining (T>N) was observed in the 32% and 44% of the cases respectively, whereas more of the 70% of the cases were positive for eIF4EP and eIF4E. Mnk1a positive staining was associated with the HER2 expression (*p* < 0.01) (Table 3). It has been previously reported that activation cascades mediated by HER2 signaling promotes the phosphorylation and translocation to the nucleus of the Y-box binding protein 1 (YB-1) factor where it binds its target genes promoting transcription, including MNK1 as a target gene [20]. MNK1b staining positively correlated with the grade and Ki-67 levels and negatively with the expression of hormone receptors. Interestingly, all the TN cases analyzed were negative for MNK1a staining while 4 of the 7 cases were positive for MNK1b. No association were found between eIF4EP or eIF4E status and the clinical-pathologic characteristics (Table 3). It is interesting to point to that the expression of MNK1b isoform was associated with eIF4EP staining (Chi-square test, *p* = 0.028).

**Table 3:** Immunohistochemical staining of MNK1 isoforms, phosphorylated eIF4E and eIF4E and clinical-pathologic characteristics

Variables	MNK1a		MNK1b		eIF4EP		eIF4E	
	T>N (%)	P^*^	T>N (% )	P^*^	T>N %	P^*^	T>N %	*P*
**Age at surgery**								
<50	4 (36)	0.703	4 (36)	1.000	8 (73)	0.676	5 (62)	0.561
>50	6 (28		9 (43)		16 (80)		14 (74)	
**pT**								
pT1	2(33)	1.000	3 (33)	0.659	5 (83)	1.000	5 (100)	0.143
pT2-T4	9(35)		13 (50)		20 (80)		15 (68)	
**Nodal status**								
Negative	4 (40)	1.000	4 (40)	1.000	9 (100)	0.136	8 (89)	0.158
Positive	6 (33)		8 (44)		13 (72)		10 (62)	
**Tumor grade**								
G1	1 (17)	0.560	2 (33)	0.026^*^	4 (67)	0.709	3 (60)	0.712
G2	5 (31)		4 (25)		12 (80)		10 (71)	
G3	5 (42)		9 (75)		10 (83)		8 (80)	
**Molecular types**								
Luminal	6 (33)	0.062	6 (33)	0.405	13 (72)	0.580	12 (75)	0.898
TN	0 (0)		4 (57)	0.041^*1^	8 (89)		6 (67)	
HER2	5 (55)		5 (55)		5 (83)		3 (75)	
**ER**								
Negative	4 (33)	1.000	8 (67)	0.010^*^	9 (82)	1.000	7 (78)	1.000
positive	7 (32)		7 (32)		17 (77)		14 (70)	
**PR**								
Negative	6 (40)	0.475	9 (60)	0.018^*^	12 (86)	0.670	9 (75)	1.000
positive	6 (30)		6 (31)		14 (74)		12 (71)	
**HER2**								
Negative	6 (25)	0.001^*^	9 (37)	0.118	18 (75)	0.642	15 (75)	0.675
positive	5 (55)		5 (55)		8 (89)		6 (67)	
**Ki-67 level**								
<15%	4 (27)	0.465	4 (27)	0.025^*^	13 (81)	1.000	10 (71)	1.000
>15%	7 (41)		10 (59)		12 (75)		11 (78)	

^*^p < 0.05 Chi-square test; ^1^relative to Luminal.

### MNK1b is associated with patient disease-free and overall survival

**Figure 3:**
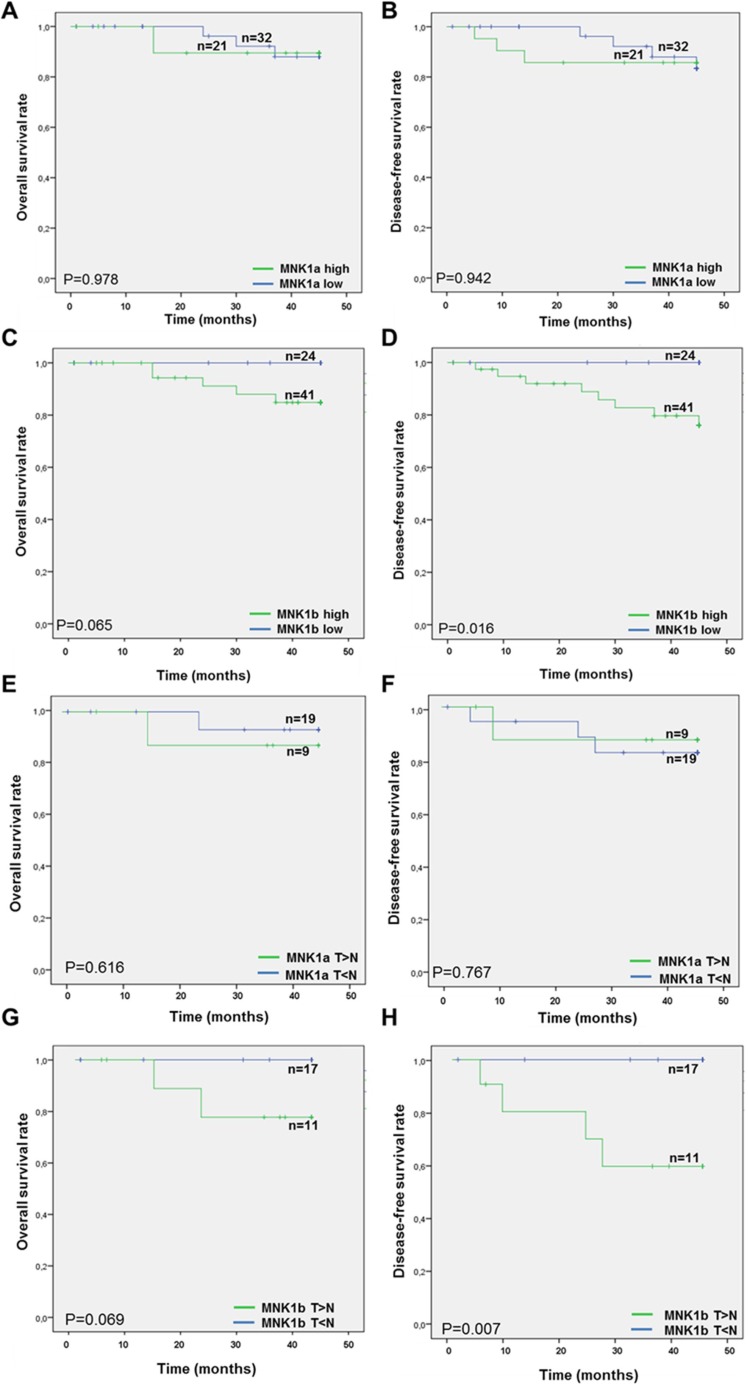
Kaplan-Meier analysis of MNK1a and MNK1b expression in breast cancer patients Patients overall survival time (**A**, **C**, **E** and **G**) and disease-free survival rate (**B**, **D**, **F** and **H**) were compared between high and low MNK1a (A–B, E–F) and high and low MNK1b (C–D, G–H) expression by Kaplan-Meier analysis. The levels of MNK1a obtained by western blot analysis (A, B) were scored in two groups considering the value of the mean (2.2, Figure 1A) as cutoff value: cases with Mnk1a levels > 2.2 constitute the high group and cases with Mnk1a levels < 2.2 constitute the low group. This method yields a survival curve that considered both outcomes (death) and censored cases (x). Significance is calculated by log-rank test between two groups. On the top of the curve “n” indicated the number of samples analyzed.

Finally, the overall and disease-free survival rates were compared among cases low or high MNK1a or MNK1b levels measured by western blot (Figure 3A–3D) or by immunohistochemistry (Figure 3E–3H), using the log rank test. MNK1a expression was not associated with overall and disease-free survival rate (Figure 3A–3B and 3E–3F) whereas the “MNK1b-high group” had lower overall and disease-free survival rates than the “MNK1b low group”, being statistically significant for disease-free survival (Figure 3D and 3H).

### The overexpression of MNK1b induces MCL1 overexpression

**Figure 4:**
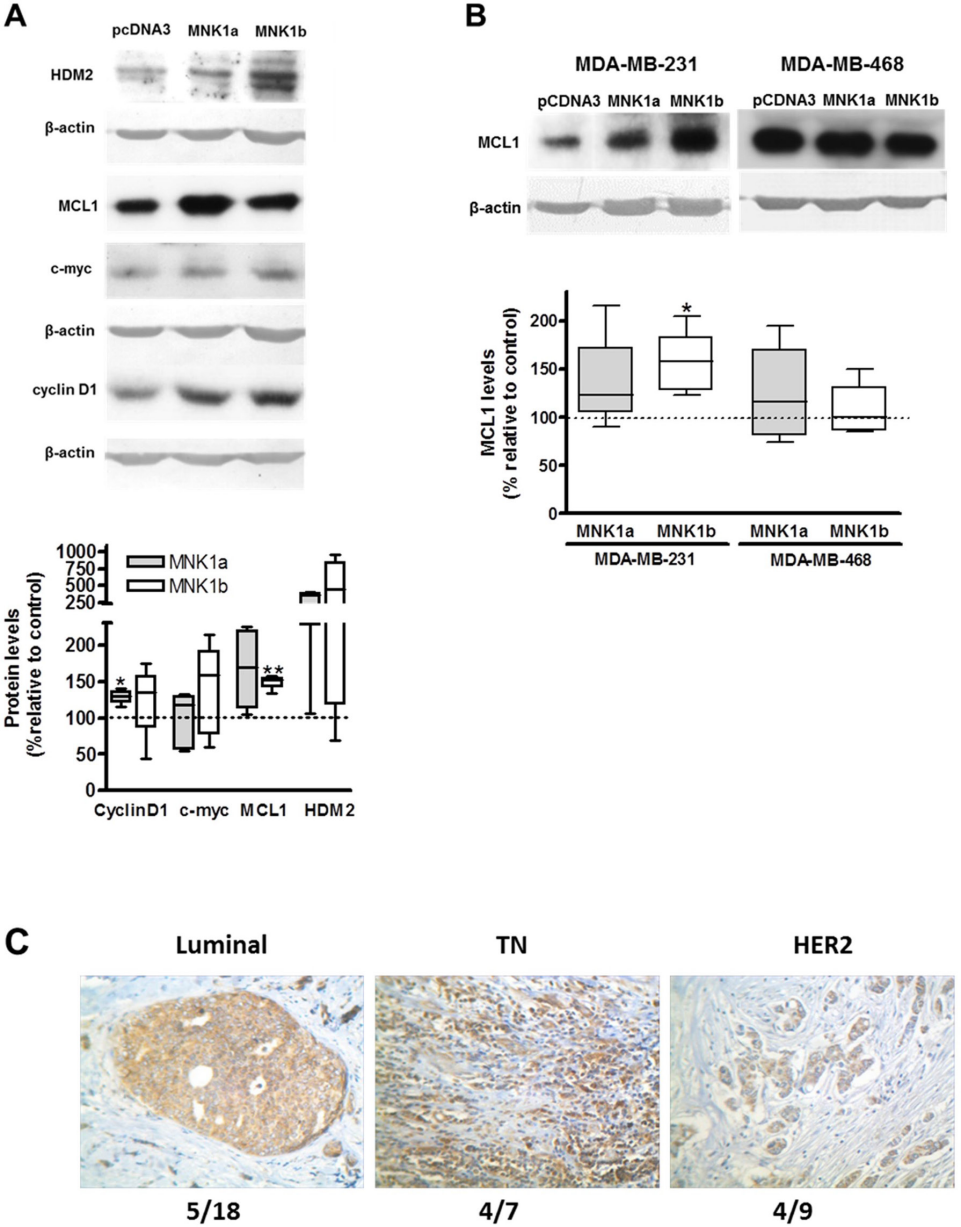
Effect of overexpression of MNK1a and MNK1b on oncoprotein levels (**A**) Lysates from MDA-MB-231 cells stably transfected with Myc-pcDNA3 (control), Myc MNK1a (MNK1a) and Myc-MNK1b (MNK1b) were subjected to SDS-PAGE 12% and western blot analysis was done as described in Materials and Methods section. Actin was used as a control for the homogeneity of loading. (**B**) MDA-MB-231 and MDA-MB-468 cells were transfected with Flag-pcDNA3 (control), Flag-pCDNA3-MNK1a (MNK1a) and Flag-pCDNA3-MNK1b (MNK1b) and after 24 hours lysed as described in Materials and Methods section. Lysates (35 µg) were subjected to SDS-PAGE 12% and western blot analysis using anti-MCL1 antibody. Actin was used as a control for the homogeneity of loading. The quantitation of the bands was normalized with respect to actin and expressed as the percentage relative to the value in control cells. The values represent the mean ± S.E.M. of 4-6 different experiments. Representative blots are shown on the top of the figure. Statistical differences relative to 100; ^*^p < 0.05; ^**^p < 0.01. (**C**) IHC staining with anti-MCL1 antibody in breast tumors. IHC was performed as described in Materials and Methods. Representative tumor sections of each molecular types are shown. Number in the bottom of the picture indicates the number of positive cases (T>N) /relative to total cases analyzed for each molecular type.

In order to study the effect of the overexpression of either MNK1a or MNK1b on tumorogenesis, we have analyzed the levels of several oncoproteins whose expression is regulated by eIF4E or by its phosphorylation, in a MNK1 or upstream MAP kinases dependent manner [12, 16, 17, 21, 22]. First, we have analyzed the expression of cyclin D1, c-myc, MCL1 and HDM2 by western blot in the stable MDA-MB-231 cells transfected with pCDNA3 (control), MNK1a (MDA-MB-MNK1a) or MNK1b (MDA-MB-MNK1b) previously generated and characterized in our laboratory [14]. As shown in Figure 4A, the overexpression of MNK1a or MNK1b induced an increase in the levels of the four proteins analyzed being statistically significant for c-myc in cells overexpressing MNK1a and for MCL1 in cells overexpressing MNK1b. As stated in [17], MNK and eIF4E phosphorylation control the translation of the antiapoptotic protein MCL1. This mechanism may contribute to the oncogenic and anti-apoptotic effects of MNK kinase. Consequently we have deeped in the study of the expression of this protein in several TN cell lines as well as in the tumor samples. The expression of MCL1 in two TN cell lines (MDA-MB-231 and MDA-MB-468) transiently transfected with MNK1a or MNK1b is showed in Figure 4B. Although total MNK1a and MNK1b (endogenous plus overexpressed levels) reached similar levels in both MNK1 overexpressing cell lines (about 3-6 fold and 20-fold relative to control MDA-MB-231 cells, respectively (Supplementary Figure 1A, 1B), we only found a statistically significant increase in the levels of MCL1 protein in MDA-MB-231 cells overexpressing MNK1b (Figure 4B).

The expression of MCL1 in paraffin tumor samples was analyzed by immunohistochemistry as described in Materials and Methods section (Figure 4C). MCL1 staining was positive in the 38% of the cases studied, being more frequent in TN (57%) than HER (44%) and Luminal (28%, *p* = 0.012).

### The overexpression of MNK1a or MNK1b induces proliferation, migration, invasion and colony formation in triple negative cell lines

**Figure 5:**
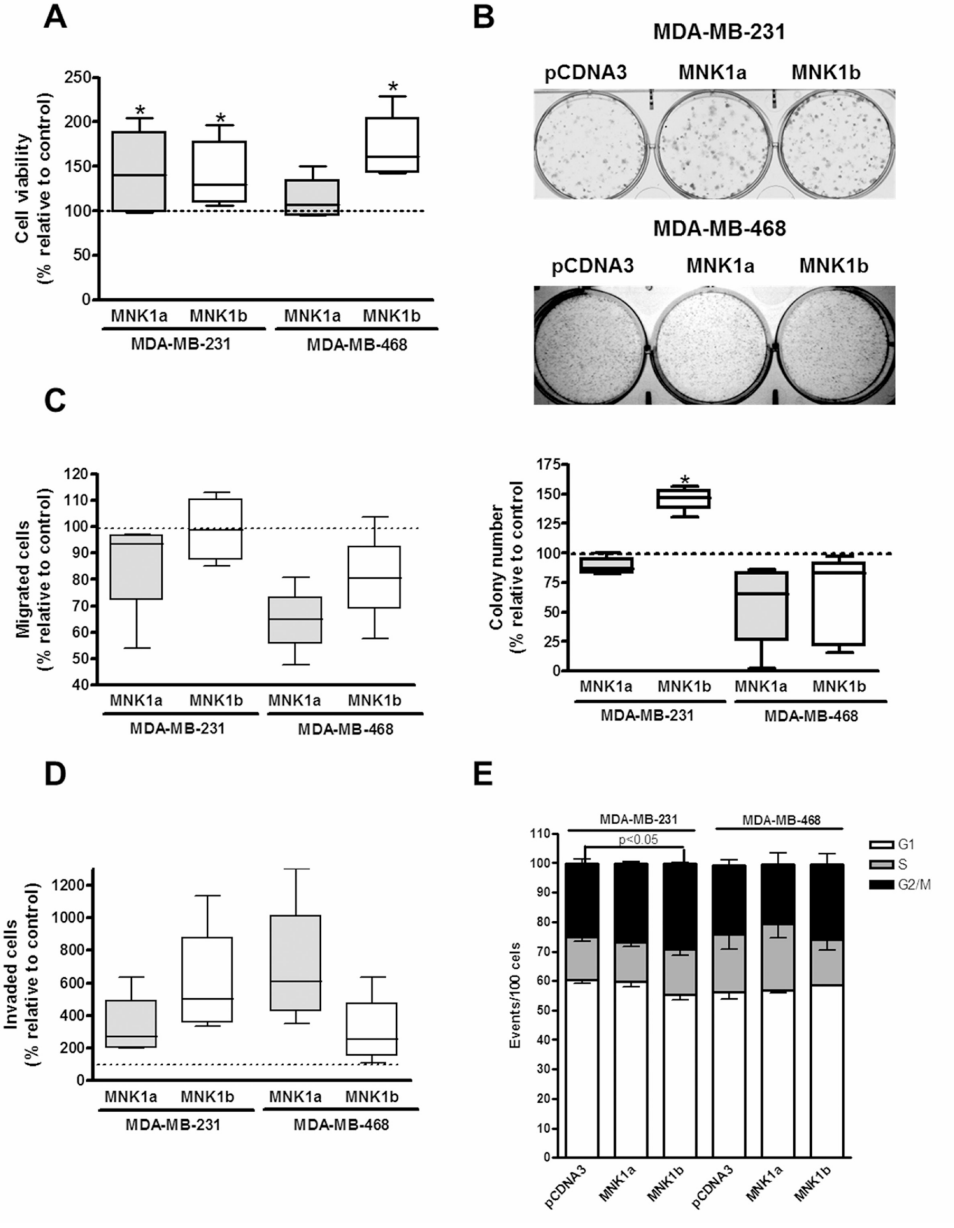
Effect of overexpression of MNK1a and MNK1b on cell viability (**A**), colony formation (**B**), migration (**C**), invasion (**D**) and cell cycle (**E**) in triple-negative breast cancer cells. MDA-MB-231 and MDA-MB-468 cells were transfected with Flag-pcDNA3 (control), Flag-pCDNA3-MNK1a (MNK1a) and Flag-pCDNA3-MNK1b (MNK1b) as described in Materials and Methods section. (A) Cell viability assays were performed after 72 h of transfection. (B) After 24 h of transfection, cells were counted and seed at 1 × 10^3^ or 5 × 10^3^ cells/well in six-well plates for MDA-MB-231 and MDA-MB-468 cells, respectively, and, after 12–14 days, the colonies were fixed, stained for 30 min with Giemsa 0.02% and counted. A transwell assay was performed withouth or with matrigel to measured migration (C) and invasion rate (D) respectively. Data are expressed as the percentage relative to the value in control cells and represent the mean ± S.E.M. of 4–6 different experiments. Statistical differences relative to 100; ^*^p < 0.05. (E) Cells were stained with PI and subjected to flow cytometry analysis. The percentage of cells gated in each phase is indicated and represent the mean ± S.E.M. of 3–4 different experiments. Statistical differences relative to the control; ^*^p < 0.05.

The effect of the overexpression of both isoforms of MNK1 on cell viability, migration, invasion, colony formation and cell cycle was studied in transienly transfected MDA-MB-231 and MDA-MB-468 cell lines as described in Materials and Methods section (Figure 5A–5E).The effect of the overexpression of MNK1 isoforms was different for the two TN cell lines analyzed. Thereby, the overexpression of MNK1b induced a substantial increase in the proliferation rate, invasion and colony formation in MDA-MB-231 cells (Figure 5A, 5B, 5D). In addition, MNK1b induced a light but significant increase in the percentage of cells in G2/M phase in this cell line (Figure 5E). However, the effect of MNK1a overexpression produced a minor effect or did not affect at these oncogenic characteristics (Figure 5). On the contrary, in MDA-MB-468 cell both MNKs did not have effect, or even induced a decrease, on the colony formation, migration and cell cycle. The effect of MNK1a overexpression on invasion was higher than MNK1b (Figure 5D) while only MNK1b induced a significant increase in the proliferation rate (Figure 5A). These results are in agreement with the lack of effect on MCL1 expression in MDA-MB-468 cells.

## DISCUSSION

Breast cancer is a complex and heterogeneous disease in terms of its histology, cellular origin, mutations, capacity to metastasize, disease progression and treatment response. Translational deregulation plays an important role in cancer, and a broad number of studies have been undertaken to characterize both the expression of translational regulators and whether these alterations are significant determinants of cancer progression (reviewed in [23]). However, although these studies include eIF4E in breast tumors [8, 24, 25] and, more recently the phosphorylation status of this protein [10, 26], very few studies have addressed the nature of MNK1/2 expression, now considered a potential target for anticancer therapy.

In this study, we determined the expression levels of MNK1a and MNK1b in tumor samples and normal adjacent tissue from breast cancer patients. In the tumor samples, significantly higher levels of both MNK1a and MNK1b protein and higher MNK1b mRNA levels were found compared to healthy tissue. Up to date, only two studies had previously determined the levels of MNK1 in tumors. In the first, the authors showed an increase in both MNK1 mRNA and protein expression in human gliomas [27]. Very recently, Hou *et al.* [28] have evaluated MNK1 expression in epithelial ovarian cancer. In this paper, the authors demonstrated a significant relationship between MNK1 expression and advanced stage and positive lymph node metastasis. In addition, high MNK1 expression indicated poor clinical outcomes in EOC tissues. However these studies did not discriminate between MNK1a and MNK1b isoforms. In our study, MNK1b levels, but not MNK1a, are associated with the patient outcomes in breast cancer. This finding indicates that MNK1b could be a novel candidate prognostic marker for use in breast cancer patients.

Interestingly, we have found that the MNK1b expression was significantly increased in triple-negative tumors, in which over 92% of the samples expressed more MNK1b in tumor tissue than in control tissue. Triple-negative tumors are characterized by the lack of expression of ER and PR and the absence of HER2 overexpression, and no targeted therapy is available for this tumor type. The expression of the antiapoptotic protein MCL1 correlates with high tumor grade and lower survival rate in breast cancer [29]. This protein plays a key role for TN cell survival [30, 31] and several recent studies indicate that could be considered as a therapy target [32–34]. It has been described that activated MNK1 promotes the onset of tumor development which was attributed to the ability of MNK1 to enhance the expression of the anti-apoptotic protein MCL1 [17]. Our cell experiments, showing that MCL1 expression upregulated by MNK1b correlates with the oncogenic characteristics of the cells, corroborate this result.

**Figure 6:**
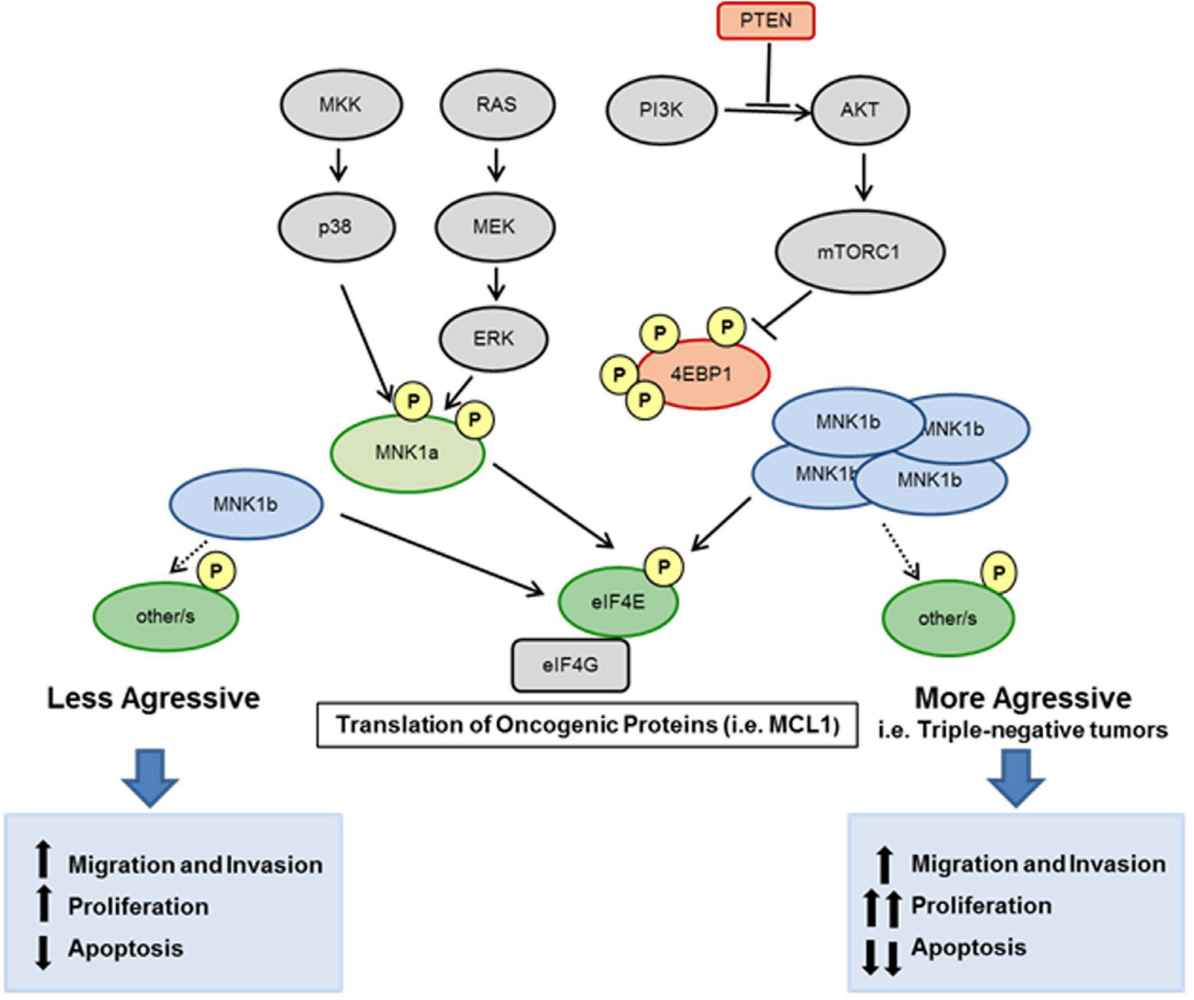
Schematic overview of this study The hyperactivation of two major signalling pathways, PI3K/AKT/mTOR and Ras/MAPK/MNK regulates eIF4E phosphorylation. MNK1a is phosphorylated by ERKs or p38 MAPKs and activates eIF4E resulting in the translation of oncogenic proteins. The isoform MNK1b is constitutively active independently of MAPKs. Overexpression of MNK1b, as occur in many of the triple-negative tumors, promote the translation of oncogenic proteins (for example, MCL1) and brings functional consequences for the cell increasing oncogenic processes as migration, invasion and proliferation and reducing apoptosis.

The hyperactivation of two major signalling pathways, PI3K/AKT/mTOR and Ras/MAPK/MNK, occurs in the majority of the cancers and eIF4E acts as a convergence point for both signalling pathways to promote tumorigenesis. PI3K activity is often high in tumors due to the loss of the PTEN tumor suppressor, which allows a constitutive activation of AKT kinase, inappropriate mTORC1 signalling, phosphorylation and inactivation of 4EBP1 and, consequenltly, eIF4E available to be phosphorylated by MNK1. This occurs in MDA-MB-468 cell line, where PTEN is mutated, and both signalling pathways are activated (Supplementary Figure 1D). In these cells, overexpressed MNK1a will be quickly activated and, plus endogenous MNK1a, will exercise its oncogenic action. In MDA-MB-231 cell line, where both pathways are less active, the overexpression of the pathways-independent isoform MNK1b promotes an increase in several oncogenic characteristic (mainly invasion and colony formation). We hypothesized that the oncogenic effect of MNK1b could produce worse progression in those tumors with non-hyperactivated AKT/mTOR or/and MAPK pathways, and, in addition, could induce resistence to therapies based on PI3K/AKT inhibitors. Altogether these findings suggest that MNK1b may be used as a therapeutic target in the future in triple-negative tumors (Figure 6).

## MATERIALS AND METHODS

### Patients

Samples and data from patients included in this study were provided by the Hospital Universitario Ramón y Cajal-IRYCIS Biobank integrated in the Spanish Platform Biobanks Network (RetBioH; www.redbiobancos.es) and were processed following standard procedures with appropriate approval of the Ethical and Scientific Committees. We analyzed 69 breast tumors and normal adjacent tissue included in OCT (optimal cutting compound) frozen via liquid nitrogen and stored at −80° C and 34 paraffin-embebed resected tumor tissues.

### Sample preparation

Eight to ten thick tissue sections (20–30 µm) were obtained using a cryostat and used for RNA and protein extraction using TRIZOL^®^ Reagent (Invitrogen, Barcelona, Spain) following the manufacturer’s protocol. In parallel, a 5-µm section was stained with hematoxylin and eosin following a standard procedure and examined under a light microscope to check the percentage of tumor present in the sample. The RNA pellet was resuspended in 50 μL of RNase-free water, treated with 1 μL of DNase I (Fermentas), quantified and stored at −80° C. The protein pellets were dissolved in 50 μL of electrophoresis buffer [180 mM Tris-HCl (pH 6.8), 9% sodium dodecyl sulphate (SDS), 6% 2-Mercaptoethanol, 15% glycerol, 0.05% bromophenol blue], heated at 95° C, cooled and stored at – 80° C.

### Immunohistochemistry

Tumor sections (5 μm) were deparaffinized and rehydrated described previously [35] and antigen retrieval was achieved by heat treatment in a pressure cooker for 2 min in 10 mM citrate buffer (pH 6.5). Next, endogenous peroxidase was blocked, and the sections were incubated either with anti-MNK1 (M-20) (1:100), anti-MNK1 (C-20) (1:100), anti-eIF4EP (1:400) and anti-eIF4E (1:150) overnight at room temperature. For the two first, the sections were incubated with MasVision anti-goat-HRP polymer kit (Master Diagnostica) for 45 min and developed with the Inmunoperoxidase DAB kit (Master Diagnostica) according to the manufacturer’s instructions. For the anti-eIF4EP and anti-eIF4E, the sections were developed using Master Polymer Plus Detection System (Peroxidase) (Master Diagnostica) according to the manufacturer’s instructions. The sections were counterstained with hematoxylin. Immunohistochemical Labeling was evaluated by four investigators (SS, MVTL, BP-M, IE-R) using uniform criteria. The pattern of staining was recorded as 0 when there was no detectable labeling, and values 1, 2, and 3 were used to indicate weak, moderate and intense labeling, respectively. Both Tumor (T) and noncancerous (N) regions were scored and the cases were classified as T>N or T<N.

### Cell culture and protein and RNA extraction

MDA-MB-231 cells (kindly provided by Dr. R. Busto (Hospital Ramón y Cajal) and last authenticated in june of 2016 using the GenePrint^®^ 10 System) were maintained in Dulbecco’s modified Eagle’s medium (DMEM)/Ham F-12 medium (PAA, Pasching, Austria) (mixed 1:1) with 10% fetal calf serum (Gibco, Grand island, NY) and 100 U/mL penicillin, 100 µg/mL streptomycin, and 25 µg/mL amphotericin (Sigma-Aldrich) in a humidified 5% CO_2_/95% air incubator at 37° C. Stable MDA-MB-231 cells [14] were maintained in the same medium with 1 mg/mL of geneticin (Invitrogen, Barcelona, Spain). MDA-MB-468 cells (kindly provided by Dr. A.M. Bajo (Universidad de Alcalá) and last authenticated in november of 2017 using the GenePrint® 10 System) were maintained in RPMI medium (PAA) with 10% fetal calf serum (Gibco, Grand island, NY) and 100 U/mL penicillin, 100 µg/mL streptomycin, and 25 µg/mL amphotericin (Sigma-Aldrich) in a humidified 5% CO_2_/95% air incubator at 37° C. To obtain lysates, the cells were mechanically harvested and washed once with cold buffer A [20 mM Tris-HCl pH 7.6, 1 mM dithiothreitol (DTT), 1 mM ethylenediaminetetraacetic acid (EDTA), 1 mM phenylmethylsulfonyl fluoride (PMSF), 1 mM benzamidine, 10 mM sodium molybdate, 10 mM sodium β-glycerophosphate, 1 mM sodium orthovanadate, 120 mM potassium chloride (KCl), 10 µg/mL antipain, 1 µg/mL pepstatin A and leupeptin]. Next, the cells were lysed in the same buffer containing 1% Triton X-100 (volume ratio 1:2) and centrifuged at 12 000 g for 10 min. Afterwards, protein concentration was determined using the BCA Kit (Pierce), and the supernatants were aliquoted and stored at −80° C until use.

### Western blot

Equal volumes of normal adjacent (control) and tumor samples were resolved in 12% SDS-PAGE gels. MNK1a and MNK1b levels were analyzed using specific anti-MNK1 antibodies from Santa Cruz Biotechnology (both 1:500 dilution); MNK1 (C-20) generated against a C-terminal peptide that only recognizes the MNK1a isoform, and MNK1 (M-20) raised against a peptide mapping to the N-terminal of MNK1. Membranes were incubated with anti-phosphorylated eIF4E antibody (Cell Signaling Technology) and reprobed with an anti-total eIF4E antibody (BD Biosciences). β-actin antibody (Sigma, St Louis, MO, USA) was used to measure housekeeping protein levels as a control to monitor the homogeneity of loading. When enough sample was available, western blot analysis was repeated loading an equal amount of actin. Two or three different western blot analyses were carried out per sample.

Cell lysates (35 μg) were resolved in 12%, 15% or 10% SDS-PAGE gels and western blots were performed as above. Actin was used as a loading control. Antibodies used in this study are listed in Supplementary Table 2.

### Quantitative real time-PCR (qRT-PCR)

The qRT-PCR reactions were performed using iScript™ One-Step Kit With SYBR^®^ Green (BioRad) in an iCycler IQ system (Bio-Rad), and the results were analyzed with iQ5 2.0 Standard Edition Optical System Software (Bio-Rad). Two primer pairs were designed (Supplementary Figure 2), one to amplify a region present in both isoforms of MNK1 (242–423), named 5′qMNK1ab (5′AGAAACAAGCAGGGCACAGT3´) and 3′qMNK1ab (5′TGCTTTTGCTTCTGGATGTG3´), and the other primer pair, named 5′qMNK1a (5′AGCATCCAGGAAG GCAAGTA3′) and 3′qMNK1a (5′ GTCCCTTTTCTGGA GCTTGC3′), only amplifies MNK1a mRNA because the 3′ primer recognizes a sequence in exon 12 that is absent in the MNK1b isoform. PGK was selected as the internal control, using the primers 5′PGK (5′ATGG ATGAGGTGGTGAAAGC3′) and 3′PGK (5′ CTTCCAGG AGCTCCAAACTG3′). For MNK1a mRNA levels, data were normalized to housekeeping mRNA levels and ΔCt (Ct_MNK1a_–Ct_housekeeping_) values were determined. ΔΔCt was calculated as ΔCt_y_ –ΔCt _control._ For MNK1b mRNA levels, ΔΔCt was calculated as (Ct_MNK1ab_ – Ct_MNK1a_)_y_ – (Ct_MNK1ab_ – Ct_MNK1a_)_control._ Y and Control are: Y = tumor sample and Control = normal adjacent tissue for tissue samples. Finally, data are expressed as 2-ΔΔCt.

### Transfection and MTT assays

MDA-MB-231 and MDA-MB-468 cells were plated at 6 × 10^3^, 3 × 10^4^ or 2.5 × 10^5^ cells/well in 96-, 24 or 6-well plates respectively. After 16–24 hours the cells were transfected with the different plasmids Flag-pCDNA3, Flag-MNK1a-pCDNA3 and Flag-MNK1b-pCDNA3 using jetPRIME transfection reagent (Polyplus Transfection) following the manufacturer’s instructions. For MTT assays, medium was removed after 72 hours and 100 μl of MTT (1 mg/ml in culture medium) was added to each well, and plates were incubated at 37° C for 3 hour. Next, 100 μl/well of lysis buffer (10% sodium dodecyl sulphate and 10 mmol/l HCl) were added and, after 24 hours of incubation, absorbance was read at 540 nm on a SpectraFluor microplate reader (TECAN). Data were calculated as the percentage relative to the cells transfected with empty vector.

### Colony-forming assays

After 16–24 hours, transfected cells as described above were collected and alive cells were counted by Trypan blue exclusion assay (Sigma-Aldrich) using the counter TC10 (Bio-Rad) and seed at 1 × 10^3^ or 5 × 10^3^ cells/well in six-well plates for MDA-MB-231 or MDA-MB-468 respectively. Approximately 12–16 days later, the colonies were fixed, stained for 30 minutes with Giemsa 0.02% (Sigma-Aldrich), and counted with a eCount Colony Counter Pen (Heathrow Scientific, Vernon Hills, IL) and a magnifying glass (×1.75) (Bel-Art Scienceware, Wayne, NJ).

### Migration and invasion assays

The migration and invasion assays were carried out using Transwell insert chambers (Corning, USA). For migration assay, cells transfected as described above, were hasterved for 16 hours, collected, counted and 4 × 10^4^ cells were plated into the upper chamber in serum-free medium. Complete medium (10% FBS) were added in the lower chamber as chemoattractant. After 24 h, medium was removed from the top and bottom of the wells; cells on the insert were fixed with 4% formaldehyde for 2 min followed by 20 min with 100% methanol. Cells were stained with 30 µM Hoechst 33342 for 15 min, protected from light, washed twice in PBS and cells on the top were removed with a cotton stick. At least five photographs were acquired for each sample using a fluorescent microscope (Olympus IX70) and the number of cells was analysed using Image J software.

For invasion assay, after starvation for 24 hours, 4 × 10^4^ cells were seeded as above into upper chambers precoated with matrigel (Corning, USA) in serum-free medium. After incubation for 48 h at 37° C, non-invading cells on the upper surface of filter were removed with cotton swabs and invading cells that migrated to the lower surface of filter were fixed, stained and scored as described above.

### Flow cytometric analysis of cell cycle

Twenty-four hours postransfection, cells were collected and fixed in 70% cold ethanol 30 min at –20° C. Afterwards, cells were washed twice with ice-cold phosphate-buffered saline (PBS) and incuabated with 50 µg/mL Propidium Iodide (Sigma) plus 100 µg/mL RNAse A (Sigma) for 1 hour at 37° C at dark. Cell cycle was analyzed by flow cytometry (FACScalibur, BD Biosciences) using selective gating to exclude doublets of cells. Cytometry data was analyzed using Flowing software 2.5.1.

### Statistics

The results are expressed as mean values ± standard deviation (SD), unless stated otherwise. Most of the samples were analyzed in triplicate. The significance relative to 1 (ratio T/NT) was analyzed by a one-sample *t*-test. For each parameter determined, the statistical significance between the two groups was analyzed with the Mann-Whitney *U* test. Three or more groups were compared by Kruskal-Wallis analysis followed by Dunn’s Multiple Comparison Test. Spearman’s correlation coefficients (r) were calculated. Pearson’s chi-square test was used to compare the difference of MNK1b-positive values among the groups. Univariate survival analyses were carried out according to Kaplan-Meier and log-rank tests. The differences were considered statistically significant when *P* values were < 0.05. The SPSS 15.0 software package was used for the statistical analysis.

## SUPPLEMENTARY MATERIALS FIGURES AND TABLES



## Abbreviations

eIF4E: eukaryotic initiation factor 4E
ER: estrogen receptor
HER2: human epidermal growth factor receptor
MNK: MAP kinase interacting kinase
PR: progesterone receptor
TN: Triple-negative

## References

[1] Campbell LL, Polyak K (2007). Breast tumor heterogeneity: cancer stem cells or clonal evolution?. Cell Cycle.

[2] Carey LA, Dees EC, Sawyer L, Gatti L, Moore DT, Collichio F, Ollila DW, Sartor CI, Graham ML, Perou CM (2007). The triple negative paradox: primary tumor chemosensitivity of breast cancer subtypes. Clin Cancer Res.

[3] Fukunaga R, Hunter T (1997). MNK1, a new MAP kinase-activated protein kinase, isolated by a novel expression screening method for identifying protein kinase substrates. EMBO J.

[4] O’Loghlen A, Gonzalez VM, Pineiro D, Perez-Morgado MI, Salinas M, Martin ME (2004). Identification and molecular characterization of Mnk1b, a splice variant of human MAP kinase-interacting kinase Mnk1. Exp Cell Res.

[5] Parra-Palau JL, Scheper GC, Wilson ML, Proud CG (2003). Features in the N and C termini of the MAPK-interacting kinase Mnk1 mediate its nucleocytoplasmic shuttling. J Biol Chem.

[6] Morley SJ, McKendrick L (1997). Involvement of stress-activated protein kinase and p38/RK mitogen-activated protein kinase signaling pathways in the enhanced phosphorylation of initiation factor 4E in NIH 3T3 cells. J Biol Chem.

[7] Thumma SC, Kratzke RA (2007). Translational control: a target for cancer therapy. Cancer Lett.

[8] De Benedetti A, Graff JR (2004). eIF-4E expression and its role in malignancies and metastases. Oncogene.

[9] Culjkovic B, Borden KL (2009). Understanding and Targeting the Eukaryotic Translation Initiation Factor eIF4E in Head and Neck Cancer. J Oncol.

[10] Fan S, Ramalingam SS, Kauh J, Xu Z, Khuri FR, Sun SY (2009). Phosphorylated eukaryotic translation initiation factor 4 (eIF4E) is elevated in human cancer tissues. Cancer Biol Ther.

[11] Adesso L, Calabretta S, Barbagallo F, Capurso G, Pilozzi E, Geremia R, Delle Fave G, Sette C (2013). Gemcitabine triggers a pro-survival response in pancreatic cancer cells through activation of the MNK2/eIF4E pathway. Oncogene.

[12] Topisirovic I, Ruiz-Gutierrez M, Borden KL (2004). Phosphorylation of the eukaryotic translation initiation factor eIF4E contributes to its transformation and mRNA transport activities. Cancer Res.

[13] Chrestensen CA, Shuman JK, Eschenroeder A, Worthington M, Gram H, Sturgill TW (2007). MNK1 and MNK2 regulation in HER2-overexpressing breast cancer lines. J Biol Chem.

[14] Garcia-Recio EM, Pinto-Diez C, Perez-Morgado MI, Garcia-Hernandez M, Fernandez G, Martin ME, Gonzalez VM (2016). Characterization of MNK1b DNA Aptamers That Inhibit Proliferation in MDA-MB231 Breast Cancer Cells. Mol Ther Nucleic Acids.

[15] Ramalingam S, Gediya L, Kwegyir-Afful AK, Ramamurthy VP, Purushottamachar P, Mbatia H, Njar VC (2014). First MNKs degrading agents block phosphorylation of eIF4E, induce apoptosis, inhibit cell growth, migration and invasion in triple negative and Her2-overexpressing breast cancer cell lines. Oncotarget.

[16] Phillips A, Blaydes JP (2008). MNK1 and EIF4E are downstream effectors of MEKs in the regulation of the nuclear export of HDM2 mRNA. Oncogene.

[17] Wendel HG, Silva RL, Malina A, Mills JR, Zhu H, Ueda T, Watanabe-Fukunaga R, Fukunaga R, Teruya-Feldstein J, Pelletier J, Lowe SW (2007). Dissecting eIF4E action in tumorigenesis. Genes Dev.

[18] Ueda T, Watanabe-Fukunaga R, Fukuyama H, Nagata S, Fukunaga R (2004). Mnk2 and Mnk1 are essential for constitutive and inducible phosphorylation of eukaryotic initiation factor 4E but not for cell growth or development. Mol Cell Biol.

[19] Wheater MJ, Johnson PW, Blaydes JP (2010). The role of MNK proteins and eIF4E phosphorylation in breast cancer cell proliferation and survival. Cancer Biol Ther.

[20] Astanehe A, Finkbeiner MR, Krzywinski M, Fotovati A, Dhillon J, Berquin IM, Mills GB, Marra MA, Dunn SE (2012). MKNK1 is a YB-1 target gene responsible for imparting trastuzumab resistance and can be blocked by RSK inhibition. Oncogene.

[21] Bilanges B, Stokoe D (2007). Mechanisms of translational deregulation in human tumors and therapeutic intervention strategies. Oncogene.

[22] Li Y, Yue P, Deng X, Ueda T, Fukunaga R, Khuri FR, Sun SY (2010). Protein phosphatase 2A negatively regulates eukaryotic initiation factor 4E phosphorylation and eIF4F assembly through direct dephosphorylation of Mnk and eIF4E. Neoplasia.

[23] Silvera D, Formenti SC, Schneider RJ (2010). Translational control in cancer. Nat Rev Cancer.

[24] Meric-Bernstam F, Chen H, Akcakanat A, Do KA, Lluch A, Hennessy BT, Hortobagyi GN, Mills GB, Gonzalez-Angulo A (2012). Aberrations in translational regulation are associated with poor prognosis in hormone receptor-positive breast cancer. Breast Cancer Res.

[25] Heikkinen T, Korpela T, Fagerholm R, Khan S, Aittomaki K, Heikkila P, Blomqvist C, Carpen O, Nevanlinna H (2013). Eukaryotic translation initiation factor 4E (eIF4E) expression is associated with breast cancer tumor phenotype and predicts survival after anthracycline chemotherapy treatment. Breast Cancer Res Treat.

[26] Ramon YCS, De Mattos-Arruda L, Sonenberg N, Cortes J, Peg V (2014). The intra-tumor heterogeneity of cell signaling factors in breast cancer: p4E-BP1 and peIF4E are diffusely expressed and are real potential targets. Clin Transl Oncol.

[27] Grzmil M, Morin P, Lino MM, Merlo A, Frank S, Wang Y, Moncayo G, Hemmings BA (2011). MAP kinase-interacting kinase 1 regulates SMAD2-dependent TGF-beta signaling pathway in human glioblastoma. Cancer Res.

[28] Hou S, Du P, Wang P, Wang C, Liu P, Liu H (2017). Significance of MNK1 in prognostic prediction and chemotherapy development of epithelial ovarian cancer. Clin Transl Oncol.

[29] Ding Q, He X, Xia W, Hsu JM, Chen CT, Li LY, Lee DF, Yang JY, Xie X, Liu JC, Hung MC (2007). Myeloid cell leukemia-1 inversely correlates with glycogen synthase kinase-3beta activity and associates with poor prognosis in human breast cancer. Cancer Res.

[30] Yang L, Perez AA, Fujie S, Warden C, Li J, Wang Y, Yung B, Chen YR, Liu X, Zhang H, Zheng S, Liu Z, Ann D (2014). Wnt modulates MCL1 to control cell survival in triple negative breast cancer. BMC Cancer.

[31] Goodwin CM, Rossanese OW, Olejniczak ET, Fesik SW (2015). Myeloid cell leukemia-1 is an important apoptotic survival factor in triple-negative breast cancer. Cell Death Differ.

[32] Braso-Maristany F, Filosto S, Catchpole S, Marlow R, Quist J, Francesch-Domenech E, Plumb DA, Zakka L, Gazinska P, Liccardi G, Meier P, Gris-Oliver A, Cheang MC (2016). PIM1 kinase regulates cell death, tumor growth and chemotherapy response in triple-negative breast cancer. Nat Med.

[33] Bai L, Zhou B, Yang CY, Ji J, McEachern D, Przybranowski S, Jiang H, Hu J, Xu F, Zhao Y, Liu L, Fernandez-Salas E, Xu J (2017). Targeted degradation of BET proteins in triple-negative breast cancer. Cancer Res.

[34] Xiao Y, Nimmer P, Sheppard GS, Bruncko M, Hessler P, Lu X, Roberts-Rapp L, Pappano WN, Elmore SW, Souers AJ, Leverson JD, Phillips DC (2015). MCL-1 Is a Key Determinant of Breast Cancer Cell Survival: Validation of MCL-1 Dependency Utilizing a Highly Selective Small Molecule Inhibitor. Mol Cancer Ther.

[35] Tejada S, Lobo MVT, Garcia-Villanueva M, Sacristan S, Isabel Perez-Morgado M, Salinas M, Elena Martin M (2009). Eukaryotic Initiation Factors (eIF) 2 alpha and 4E Expression, Localization, and Phosphorylation in Brain Tumors. Journal of Histochemistry & Cytochemistry.

