# Supplementary material for: Increased expression of MNK1b, the spliced isoform of MNK1, predicts poor prognosis and is associated with triple-negative breast cancer
Source: Oncotarget. 2018 Feb 5;9(17):13501–16. doi: 10.18632/oncotarget.24417 (PMC5862594; doi:10.18632/oncotarget.24417)
Supplement: Supplementary file 1 [file oncotarget-09-13501-s001.pdf]

Increased expression of MNK1b, the spliced isoform of MNK1, predicts poor prognosis and is associated with triple-negative breast cancer

SUPPLEMENTARY MATERIALS

Supplementary Table 1: Levels of MNK1 isoforms, phosphorylated eIF4E and eIF4E and clinical-pathologic characteristics

Variables	MNK1a	MNK1b	eIF4EP	eIF4E
**Age at surgery**	mean ± S.D. (*n*)	mean ± S.D. (*n*)	mean ± S.D. (*n*)	mean ± S.D. (*n*)
<50	2.17 ± 1.99 (15)	6.28 ± 10.15 (10)	1.74 ± 1.35 (17)	2.73 ± 1.83 (16)
>50	2.42 ± 2.11 (39)	4.92 ± 4.96 (25)	1.35 ± 1.03 (37)	2.53 ± 1.79 (38)
**pT**				
pT1	1.91 ± 1.81 (15)	10.19 ± 10.23(10)*	1.21 ± 0.75 (11)	1.97 ± 0.88 (14)
pT2-4	2.52 ± 2.19 (38)	3.60 ± 3.31 (24)	1.63 ± 1.22 (42)	2.78 ± 1.98 (20)
**Nodal status**				
Negative	2.26 ± 1.87 (20)	6.48 ± 5.95 (13)	1.74 ± 1.33 (18)	1.69 ± 1.27 (17)**
Positive	2.52 ± 2.31 (30)	5.08 ± 7.64 (19)	1.43 ± 1.05 (33)	3.02 ± 1.90 (35)
**Tumor grade**				
G1	1.31 ± 1.10 (8)	9.39 ± 13.09 (6)	1.65 ± 1.46 (8)	2.18 ± 1.43 (7)
G2	2.69 ± 2.43 (25)	5.63 ± 5.24 (16)	1.43 ± 0.83 (26)	2.75 ± 1.64 (27)
G3	2.25 ± 1.80 (22)	3.22 ± 2.75 (14)	1.69 ± 1.38 (19)	2.51 ± 211 (20)
**Molecular types**				
Luminal	1.98 ± 1.74 (32)	6.42 ± 7.78 (24)	1.40 ± 0.94 (33)	2.54 ± 1.60 (29)
TN	2.99 ± 2.76 (12)	2.86 ± 2.03 (4)	1.88 ± 1.08 (9)	2.50 ± 2.42 (11)
HER2	2.54 ± 2.08 (11)	3.25 ± 2.38 (8)	1.63 ± 1.63 (12)	2.66 ± 1.68 (15)
**ER**				
Negative	2.88 ± 2.49 (20)	3.33 ± 215 (11)	1.68 ± 1.23 (17)	2.50 ± 2.13 (20)
positive	1.99 ± 1.73 (35)	6.19 ± 7.60 (25)	1.46 ± 1.10 (37)	2.61 ± 1.57 (35)
**PR**				
Negative	3.03 ± 2.33 (22)	3.74 ± 2.87 (12)	1.66 ± 1.13 (20)	2.65 ± 2.21 (23)
positive	1.83 ± 1.75 (33)*	6.11 ± 7.79 (24)	1.45 ± 1.15 (34)	2.51 ± 1.41 (32)
**HER2**				
Negative	2.26 ± 2.08 (44)	5.96 ± 7.32 (28)	1.51 ± 0.97 (42)	2.53 ± 1.83 (40)
positive	2.54 ± 2.08 (11)	3.26 ± 2.38 (8)	1.63 ± 1.63 (12)	2.66 ± 1.68 (15)
**Ki-67 level**				
<15%	2.11 ± 2.10 (22)	7.25 ± 8.73 (17)	1.51 ± 110 (23)	2.77 ± 1.77 (25)
>15%	2.54 ± 2.09 (31)	3.75 ± 3.28 (18)	1.61 ± 1.20 (29)	2.44 ± 1.84 (28)

*$p < 0.05$ Mann-Whitney U test.

Supplementary Table 2: Antibodies used in this study

Antibody	Source	Catalog number	Dilution
MNK1 (C-20)	Santa Cruz Biotechnology	sc-6965	1:500
MNK1 (M-20)	Santa Cruz Biotechnology	sc-6962	1:500
eIF4E(Ser209)P	Abcam	Ab76256	1:2000
eIF4E	BD Biosciences	610269	1:2000
Actin	Sigma Aldrich	A5441	1:5000
c-myc (9E10)	Santa Cruz Biotechnology	sc-40	1:500
MCL-1 (S-19)	Santa Cruz Biotechnology	sc-819	1:200
HMD2	Santa Cruz Biotechnology	sc-965	1:500
Cyclin-D1	Sigma Aldrich	C7464	1:500
AKT(Ser473)P	Cell Signaling	9271S	1:2000
AKT	Cell Signaling	9272S	1:1000
ERKdiP	Sigma	M9692	1:5000
ERK1/2	Sigma	M5670	1:5000
4EBP1	Cell Signaling	9452S	1:1000

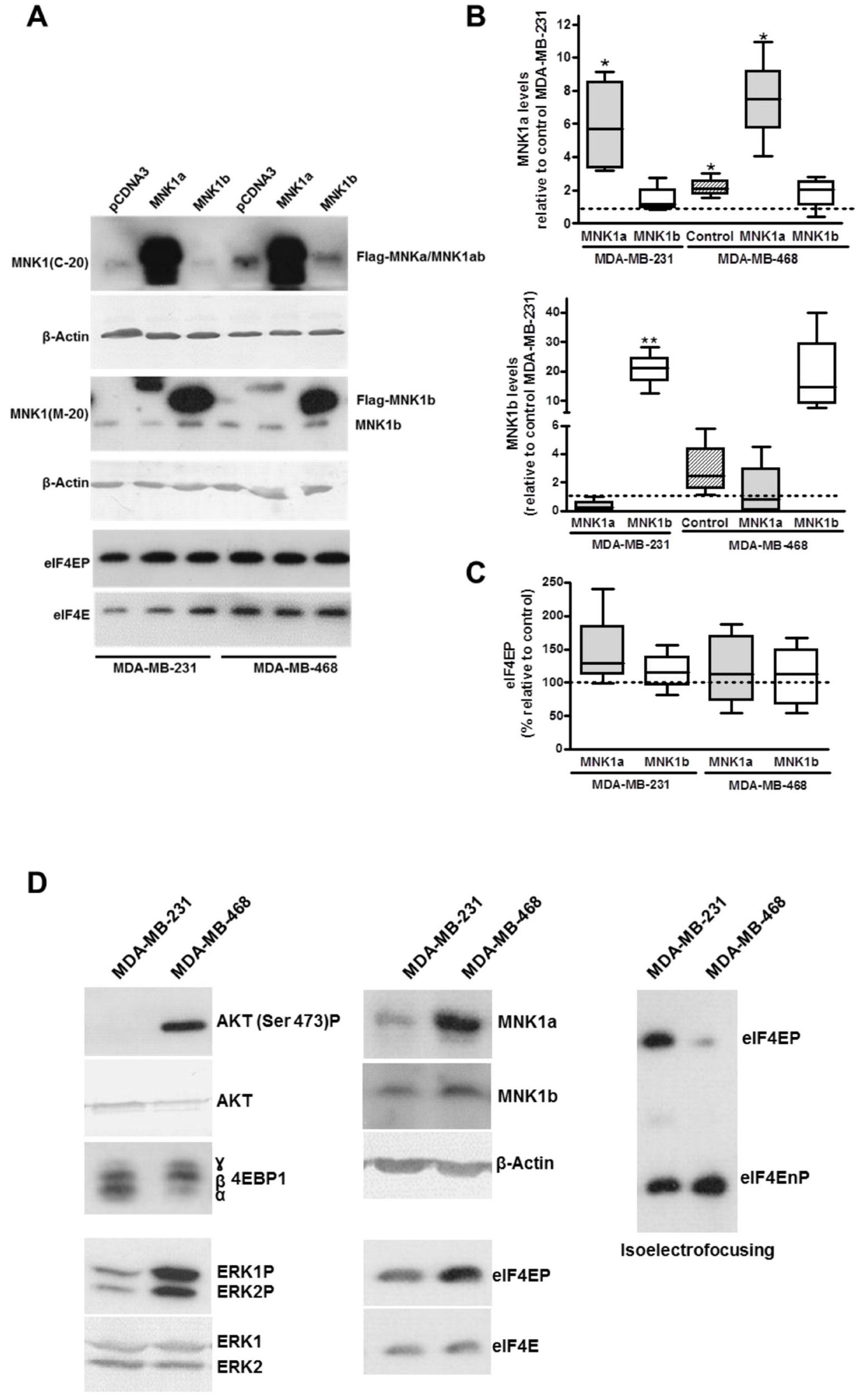

Supplementary Figure 1: (**A**) MDA-MB-231 and MDA-MB-468 cells were transfected with Flag-pcDNA3 (control), Flag-pCDNA3-MNK1a (MNK1a) and Flag-pCDNA3-MNK1b (MNK1b) and after 24 hours lysed as described in Materials and Methods section. Lysates (35 µg) were subjected to SDS-PAGE 12% and western blot analysis using anti-MNK1 (C-20), MNK1 (M-20), eIF4E(Ser209)P and eIF4E antibodies. Actin was used as a control for the homogeneity of loading. (**B**) The quantitation of the bands was normalized with respect to actin and expressed as the ratio between MNK1a or MNK1b transfected and control MDA-MB-231 cells. The values represent the mean ± S.E.M. of 4 different experiments. Statistical differences relative to 1; $^{*}p < 0.05$; $^{**}p < 0.01$. (**C**) The ratio between eIF4EP and eIF4E was calculated and expressed as the percentage relative to the value in control cells. The values represent the mean ± S.E.M. of 4-5 different experiments. (**D**) Lysates (35 µg) were subjected to SDS-PAGE 15% (for 4EBP1) or 12% and western blot analysis using specific antibodies (listed in Supplementary Table 2). Actin was used as a control for the homogeneity of loading. Right panel: Isoelectrofocusing was performed as described in [4].

```
MNK1a     ATGGTATCTTCTCAAAAGTTGGAAAAACCTATAGAGATGGGCAGTAGCGAACCCCTTCCC 60
MNK1b     ATGGTATCTTCTCAAAAGTTGGAAAAACCTATAGAGATGGGCAGTAGCGAACCCCTTCCC 60
                         Exón 1
MNK1a     ATCGCAGATGGTGACAGGAGGAGGAAGAAGAAGCGGAGGGGCCGGGCCACTGACTCCTTG 120
MNK1b     ATCGCAGATGGTGACAGGAGGAGGAAGAAGAAGCGGAGGGGCCGGGCCACTGACTCCTTG 120
                                            Exón 2
MNK1a     CCAGGAAAGTTTGAAGATATGTACAAGCTGACCTCTGAATTGCTTGGAGAGGGAGCCTAT 180
MNK1b     CCAGGAAAGTTTGAAGATATGTACAAGCTGACCTCTGAATTGCTTGGAGAGGGAGCCTAT 180

MNK1a     GCCAAAGTTCAAGGTGCCGTGAGCCTACAGAATGGCAAAGAGTATGCCGTCAAAATCATC 240
MNK1b     GCCAAAGTTCAAGGTGCCGTGAGCCTACAGAATGGCAAAGAGTATGCCGTCAAAATCATC 240
                         Exón 3
MNK1a     GAGAAACAAGCAGGGCACAGTCGGAGTAGGGTGTTTCGAGAGGTGGAGACGCTGTATCAG 300
MNK1b     GAGAAACAAGCAGGGCACAGTCGGAGTAGGGTGTTTCGAGAGGTGGAGACGCTGTATCAG 300
                                         Exón 4
MNK1a     TGTCAGGGAAACAAGAACATTTTGGAGCTGATTGAGTTCTTTGAAGATGACACAAGGTTT 360
MNK1b     TGTCAGGGAAACAAGAACATTTTGGAGCTGATTGAGTTCTTTGAAGATGACACAAGGTTT 360
                                                     Exón 5
MNK1a     TACTTGGTCTTTGAGAAATTGCAAGGAGGTTCCATCTTAGCCCACATCCAGAAGCAAAAG 420
MNK1b     TACTTGGTCTTTGAGAAATTGCAAGGAGGTTCCATCTTAGCCCACATCCAGAAGCAAAAG 420

MNK1a     CACTTCAATGAGCGAGAAGCCAGCCGAGTGGTGCGGGACGTTGCTGCTGCCCTTGACTTC 480
MNK1b     CACTTCAATGAGCGAGAAGCCAGCCGAGTGGTGCGGGACGTTGCTGCTGCCCTTGACTTC 480
                                         Exón 6
MNK1a     CTGCATACCAAAGGCATTGCTCATCGTGATCTGAAACCAGAAAATATATTGTGTGAATCT 540
MNK1b     CTGCATACCAAAGGCATTGCTCATCGTGATCTGAAACCAGAAAATATATTGTGTGAATCT 540
                                                  Exón 8
MNK1a     CCAGAAAAGGTGTCTCCAGTGAAAATCTGTGACTTTGACTTGGGCAGTGGGATGAAACTG 600
MNK1b     CCAGAAAAGGTGTCTCCAGTGAAAATCTGTGACTTTGACTTGGGCAGTGGGATGAAACTG 600

MNK1a     AACAACTCCTGTACCCCCATAACCACACCAGAGCTGACCACCCCATGTGGCTCTGCAGAA 660
MNK1b     AACAACTCCTGTACCCCCATAACCACACCAGAGCTGACCACCCCATGTGGCTCTGCAGAA 660
                    Exón 9
MNK1a     TACATGGCCCCTGAGGTAGTGGAGGTCTTCACGGACCAGGCCACATTCTACGACAAGCGC 720
MNK1b     TACATGGCCCCTGAGGTAGTGGAGGTCTTCACGGACCAGGCCACATTCTACGACAAGCGC 720

MNK1a     TGTGACCTGTGGAGCCTGGGCGTGGTCCTCTACATCATGCTGAGTGGCTACCCACCCTTC 780
MNK1b     TGTGACCTGTGGAGCCTGGGCGTGGTCCTCTACATCATGCTGAGTGGCTACCCACCCTTC 780
                                       Exón 10
MNK1a     GTGGGTCACTGCGGGGCCGACTGTGGCTGGGACCGGGGCGAGGTCTGCAGGGTGTGCCAG 840
MNK1b     GTGGGTCACTGCGGGGCCGACTGTGGCTGGGACCGGGGCGAGGTCTGCAGGGTGTGCCAG 840

MNK1a     AACAAGCTGTTTGAAAGCATCCAGGAAGGCAAGTATGAGTTTCCTGACAAGGACTGGGCA 900
MNK1b     AACAAGCTGTTTGAAAGCATCCAGGAAGGCAAGTATGAGTTTCCTGACAAGGACTGGGCA 900

MNK1a     CACATCTCCAGTGAAGCCAAAGACCTCATCTCCAAGCTCCTGGTGCGAGATGCAAAGCAG 960
MNK1b     CACATCTCCAGTGAAGCCAAAGACCTCATCTCCAAGCTCCTGGTGCGAGATGCAAAGCAG 960
                                    Exón 11
MNK1a     AGACTTAGCGCCGCCCAAGTTCTGCAGCACCCATGGGTGCAGGGGCAAGCTCCAGAAAAG 1020
MNK1b     AGACTTAGCGCCGCCCAAGTTCTGCAGCACCCATGGGTGCAGGGG--------------- 1005

MNK1a     GGACTCCCCACGCCGCAAGTCCTCCAGAGGAACAGCAGCACAATGGACCTGACGCTCTTC 1080
MNK1b     -----------------------------GAACAGCAGCACAATGGACCTGACGCTCTTC 1036
                    Exón 12
MNK1a     GCAGCTGAGGCCATCGCCCTTAACCGCCAGCTATCTCAGCACGAAGAGAACGAACTAGCA 1140
MNK1b     GCAGCTGAGGCCATCGCCCTTAACCGCCAGCTATCTCAGCACGAAGAGAACGAACTAGCA 1096

MNK1a     GAGGAGCCAGAGGCACTAGCTGATGGCCTCTGCTCCATGAAGCTTTCCCCTCCCTGCAAG 1200
MNK1b     GAGGAGCCAGAGGCACTAGCTGATGGCCTCTGCTCCATGAAGCTTTCCCCTCCCTGCAAG 1156
                                                  Exón 13
MNK1a     TCACGCCTGGCCCGGAGACGGGCCCTGGCCCAGGCAGGCCGTGGTGAAGACAGGAGCCCG 1260
MNK1b     TCACGCCTGGCCCGGAGACGGGCCCTGGCCCAGGCAGGCCGTGGTGAAGACAGGAGCCCG 1216

MNK1a     CCCACAGCACTCTGA 1275
MNK1b     CCCACAGCACTCTGA 1231
```

Supplementary Figure 2: Comparison of nucleotides sequences of MNK1a and MNK1b. 5′qMNK1ab and 3′qMNK1ab primers (green) amplify both mRNAs, 5′qMNK1a and 3′qMNK1a (blue) amplify MNK1a mRNA. The termination codon for MNK1b is indicated in a red box.
